# The Medical Examination of Recruits

**Published:** 1915-12-25

**Authors:** 


### THE MEDICAL EXAMINATION OF RECRUITS.

Haste and thoroughness are not easily reconciled with one another in many undertakings, and most certainly they cannot be combined in the process of medical examination. The difficulty is all the greater in proportion as the individual examined is free from evidence of disease. A flagrant defect may be discovered at a glance, but to prove complete soundness demands time, for it means the establishment of a universal negative. To put the position in other words, the certification of an individual as a person in perfect health is the most difficult of all diagnoses. When this is remembered there can be no surprise that mistakes are made by medical officers engaged in the examination of recruits. Especially must this be true of such work when it is necessarily conducted under the conditions which have existed during the last few weeks. Neither time nor other circumstances have been suitable to careful observation or to nicety of judgment. In which direction the mistake is made will depend very largely on the temperament of the examiner. While one will be charitable in his interpretation another will be severe, and hence we hear both of acceptances where rejection should have been the verdict and of exclusions where admission should have carried the day. Hurried critics, on the basis of such experiences, rush into print with comments on the incapacity of the medical profession. On their own incapacity to appreciate the difficulties of the situation they are not well informed.

One of the stones of stumbling has undoubtedly been the existence of cardiac murmurs and irregularities. Certainly until within comparatively recent years such clinical facts were, according to the then current teaching, regarded as grounds for anxiety and even for alarm. The student was trained to regard both the one and the other as a final and conclusive proof of a damaged heart, and to shape his prognosis on this conclusion. In the highly practical field of life insurance, also, these events were judged as of sinister meaning, and they made for heavy premiums or even for absolute exclusion. So far as professional judgment has been modified this result has been due to a more precise analysis based very largely on graphic methods of investigation. From these has arisen a capacity for differentiation and for the recognition of those irregular actions of the heart which are physiological, as contrasted with those which mean diseased cardiac structure. Now it must be admitted that a knowledge of these methods and of the conclusions which spring from them have not yet penetrated the whole body of the profession. Further, even for those who have knowledge on these points, some skill and care in investigation are necessary to a confident conclusion. Can such qualifications be expected when recruits and recruiting officers are alike clamouring for dispatch? It is one thing for the physician in the quiet and seclusion of his consulting-room to dismiss a cardiac murmur or irregularity as unimportant, but the problem has a different face to the medical officer pressed by a small army of excited men. In these latter circumstances it is idle to call for fine distinctions and nicely adjusted decisions. Mistakes then are inevitable, and if their final consequences are to be avoided a doubtful issue must be carried to a skilled and leisured court of appeal. If this were provided we have no doubt that a certain number of men rejected for heart disease would find their way into the ranks and would prove the justness of the considered verdict. But in the absence of such a tribunal more or less rough conclusions are alone possible, and individuals ought not to be blamed for what is inherent in the circumstances.

